# Inhibition of Histone H3K18 Acetylation-Dependent Antioxidant Pathways Involved in Arsenic-Induced Liver Injury in Rats and the Protective Effect of *Rosa roxburghii Tratt* Juice

**DOI:** 10.3390/toxics11060503

**Published:** 2023-06-03

**Authors:** Lu Ma, Teng Hou, Kai Zhu, Aihua Zhang

**Affiliations:** The Key Laboratory of Environmental Pollution Monitoring and Disease Control, Ministry of Education, Department of Toxicology, School of Public Health, Guizhou Medical University, Guiyang 550025, China; malu229@126.com (L.M.); hou.teng@hotmail.com (T.H.); 18275101004@163.com (K.Z.)

**Keywords:** arsenic, liver injury, oxidative damage, H3K18ac, heat stock protein, *Rosa roxburghii Tratt* juice

## Abstract

Arsenic is a common environmental toxicant. Long-term arsenic exposure can induce various types of liver injury, but the underlying mechanism remains unclear, so effective prevention and treatment measures are unknown. This study aims to explore the mechanism of arsenic-induced rat liver injury based on the histone H3K18 acetylation-dependent antioxidant pathway and to identify the role of a medicinal and edible resource, *Rosa roxburghii Tratt* juice, in combating it. Hepatic steatosis and inflammatory cell infiltration were observed in rats exposed to different doses of NaAsO_2_ using histopathological measurement. Increased 8-OHdG and MDA in liver tissue corroborated hepatic oxidative damage. We further found that a reduction in H3K18ac in the liver showed a dose–response relationship, with an increase in the NaAsO_2_ treatment dose, and it was remarkably associated with increased 8-OHdG and MDA. The results of ChIP-qPCR identified that the decreased enrichment of H3K18ac in promoters of the *Hspa1a* and *Hspb8* genes culminated in the inhibition of the genes’ expression, which was found to be involved in the aggravation of hepatic oxidative damage induced by arsenic. Notably, *Rosa roxburghii Tratt* juice was found to reduce 8-OHdG and MDA in the liver, thereby alleviating the histopathological lesions induced by arsenic, which was modulated by recovering the H3K18ac-dependent transcriptional activation of the *Hspa1a* and *Hspb8* genes. Taken together, we provide a novel epigenetics insight into clarifying the mechanism of arsenic-induced liver injury and its rescue by *Rosa roxburghii Tratt* juice.

## 1. Introduction

Environmental arsenic contamination is one of the major global public health problems [1]. Contaminated water, food and air are the main routes of arsenic exposure. Long-term exposure to arsenic can harm multiple organs such as the skin, liver, lung and nervous system, and it can even induce tumors [2]. The liver, as the main organ of arsenic metabolism, is also a major target organ for toxicity from arsenic exposure [3]. Epidemiological studies have shown that chronic arsenic exposure can increase the risk of liver lesions, such as liver dysfunction [4,5], noncirrhotic intrahepatic portal hypertension [6] and cirrhosis [7]. The animal models demonstrated that arsenic could induce liver inflammation [8] and liver fibrosis [9]. Reactive oxygen species (ROS) and their mediated oxidative damage are the common denominators in arsenic pathogenesis and are also considered as the initial event of arsenic-induced liver injury [10,11]. Hepatocyte inflammation [12] and hepatic stellate cell activation [13] induced by arsenic can be triggered by superoxide accumulation. Continuous antioxidant intervention has been reported to be a potential strategy via which to antagonize arsenic hepatotoxicity [14,15]. Therefore, exploring the mechanism of arsenic-induced hepatic oxidative damage, as well as identifying available antioxidant agents on this basis, is very important for the prevention of arsenic-induced liver injury.

Emerging evidence suggests that epigenetic modification may be an important regulator of arsenic-induced oxidative damage. Aberrant epigenetic modifications, such as DNA methylation [16,17] and miRNAs [18,19], are involved in the oxidation–antioxidant imbalance induced by arsenic, by dysregulating the expression of oxidative-stress-related genes (OSR). Additionally, the reversibility of epigenetic modification provides new insight into disease therapy based on antioxidant strategies. Many natural antioxidants, including curcumin [20] and resveratrol [21], have been found to attenuate oxidant–antioxidant imbalance by correcting aberrant epigenetic modifications. Histone modification, one of the patterns of epigenetic modification, has been reported to regulate oxidative stress in organ inflammation [22,23]. Histone modifications, such as H4K16ac [24] and H3K79me1 [25], were revealed to respond to arsenic exposure and be involved in arsenic toxicity. Our previous study found that H3K18ac might be a reliable epigenetic regulator of arsenic-induced oxidative damage. The results showed that decreased levels of H3K18ac were significantly associated with the increased burden of oxidative damage in the arsenic poisoning population, and the models of skin and embryonic kidney epithelial cells with arsenic treatment confirmed that the H3K18ac-dependent transcription activation of OSR genes, including *GCLC*, *HSP90AA1*, *NCF2,* etc., could act against arsenic-induced oxidative stress [26]. The results provide a useful target for us to further explore the mechanism of arsenic-induced oxidative damage in target organs. However, whether and how H3K18ac is involved in hepatic oxidative damage induced by arsenic is still unclear.

As mentioned above, our previous study suggested that heat shock proteins might be one of the targets of H3K18ac in the regulation of arsenic-induced oxidative damage. Heat shock proteins (HSPs) are molecular chaperones, involved in the maintenance of cellular homeostasis through the initiation of protein folding, or the repair or degradation of irreparable proteins. HSPs play an important role in the inhibition or neutralization of excessive ROS and in maintaining oxidation–antioxidant balance by cooperating with antioxidant enzymes [27]. The redox imbalance caused by the dysregulation of heat shock protein expression was demonstrated to be one of the critical mechanisms involved in liver lesions [28,29]. Moreover, heat shock proteins have been found to be involved in the regulation of oxidative damage induced by environmental pollutants such as cadmium [30] and arsenic [31]. Therefore, HSPs were targeted to explore the mechanism of H3K18ac in regulating arsenic-induced hepatic oxidative damage.

Another major goal of this study is to identify effective intervention agents. *Rosa roxburghii Tratt* (*R. roxburghii*) is a medicinal and edible resource. In recent years, *R. roxburghii* has gained attention on account of its numerous beneficial antioxidant, antimutation, and radioprotection properties, as well as its effects against diseases such as cancer and dyslipidemia [32]. Our previous study found that *R. roxburghii* juice could antagonize arsenic-induced oxidative damage in rat liver and alleviate liver dysfunction [33]. Nevertheless, the role and mechanism of *R. roxburghii* juice in antagonizing hepatic oxidative damage and subsequent histopathological lesions are unclear, which could discourage the application of *R. roxburghii* in the prevention of liver injury caused by arsenic.

In this study, we constructed a rat liver injury model induced by subchronic arsenic exposure and explored the mechanism of H3K18ac in arsenic-induced hepatic oxidative damage by evaluating the role of H3K18ac in the transcription regulation of HSP genes. Additionally, we performed an intervention with *R. roxburghii* juice and identified its role and mechanism in antagonizing arsenic-induced liver injury based on the H3K18ac–HSPs axis-dependent antioxidant pathway.

## 2. Materials and Methods

### 2.1. Animal Model

Forty-eight healthy Wistar rats, with initial weights of 80 g–100 g, were randomly divided into six groups, with eight rats in each group, half male and half female. Six groups were treated with sodium arsenite (NaAsO_2_) (0.0, 2.5, 5.0, 10.0 mg/kg) (Sigma, St. Louis, MO, USA), 10 mL/kg of *R. roxburghii* juice (Sinopharm Group Guizhou Healthcare Industry Development Co., Ltd., Guiyang, China; the health food license number of National Health Commission of the People’s Republic of China [2002]0004) and 10.0 NaAsO_2_ mg/kg + 10 mL/kg of *R. roxburghii* juice by gavage, respectively, once a day for 16 weeks. All animals were given a standard diet and housed in separate cages with a temperature of 22–24 °C, humidity of 60–70%, and light/dark for 12 h/12 h. The rats were anesthetized using an intraperitoneal injection of 1% sodium pentobarbital at the end of the treatment and then sacrificed by cervical dislocation. The liver tissues of the rats were collected. A portion of the isolated liver was soaked in 4% paraformaldehyde for 48 h, and then hematoxylin and eosin (HE) staining were performed to assess the pathological changes. The remaining isolated liver was stored at −80 °C for subsequent assays. The study protocol was reviewed and approved by the Ethics Committee of Guizhou Medical University (Approval No. 1403059).

### 2.2. Arsenic Concentration in Liver

The liver tissue of the rats was digested using concentrated nitric acid and hydrogen peroxide via microwave digestion (Thermo Fisher, Waltham, MA, USA), and then was redissolved in 2% HNO_3_. The contents of arsenic in the rat liver samples were analyzed with inductively coupled plasma mass spectrometry (ICP-MS) (Thermo Fisher, Waltham, MA, USA). The relative concentration of arsenic in the liver was standardized by the weight of the digested liver tissue and expressed as μg/g liver.

### 2.3. Analysis of Liver Oxidative Damage

8-hydroxylated deoxyguanosine (8-OHdG) and malondialdehyde (MDA) were measured in isolated liver from the rats to assess the hepatic oxidative damage. In total, 50 mg of liver tissue was ground into a liver homogenate with PBS buffer, and then the supernatant was separated by centrifuging at 600 g/min for 20 min. A competition enzyme-linked immunosorbent assay and thiobarbituric acid method were used to measure the contents of 8-OHDG and MDA in the supernatant, respectively, according to the manufacturer’s instructions for the Rat 8-OHdG Elisa Kit and Lipid Peroxidation MDA Assay Kit (Beyotime, Shanghai, China).

### 2.4. Analysis of H3K18ac Levels in Liver

Histones were extracted from the isolated liver of rats via acid extraction, and the level of H3K18ac was measured by using a sandwich enzyme-linked immunosorbent assay (ELISA). The detailed protocol was as described previously [26]. The antibodies used in the assay included the coated antibody H3-C (Sigma, St. Louis, MO, USA), the target primary antibodies H3 and H3K18ac (Abcam, Cambridge, MA, USA), and the anti-rabbit secondary antibody (Abcam, Cambridge, MA, USA). The relative concentration of H3K18ac was calculated using the standard curves configurated by recombinant H3K18ac (Active Motif, Carlsbad, CA, USA), and was standardized by the weight of the liver tissue used for histone extraction.

### 2.5. Selection of Representative Heat Shock Proteins

The gene expression profiles (GSE19662) based on the GPL570 platform were downloaded from the Gene Expression Omnibus (GEO) database (http://www.ncbi.nlm.nih.gov/geo/ accessed on 5 June 2019). The data were obtained from 12 samples of rat primary hepatocytes with 0, 0.1, 0.3, and 1 ppm of NaAsO_2_ treatment, respectively. The differentially expressed genes (DEGs) were extracted by comparing the controls and arsenic-exposed samples with the threshold as a corrected *p*-value < 0.05. Log fold-changes (FC) ≤ 0.63 or ≥1.1. 9 DEGs were obtained, including *Hsp90aa1*, *Hsp90ab1*, *Hspa1a*, *Hspb1*, *Hspb8*, *Hsph1*, *DNAJB1*, *DNAJA4*, and *DNAJC5*, which were the representative genes responding to arsenic exposure.

To obtain the genes involved in oxidative stress, the keyword “Oxidative stress” was used to search in the GeneCards database (https://www.genecards.org accessed on 5 June 2019), and 9555 genes were grabbed. In total, 56 HSP genes, which were potentially the representative genes involved in oxidative stress, were screened from them. Then, 5 overlapping HSP genes were identified from the above two data points of representative genes using the online Venn analysis tool (https://www.omicstudio.cn/tool/6 accessed on 5 June 2019), including *Hsp90aa1, Hsp90ab1, Hspa1a, Hspb1*, and *Hspb8*, which were included in the subsequent study as the final representative HSP genes.

### 2.6. Quantitative Real-Time PCR

Total RNA was isolated from liver tissue. A Prime Script TM RT reagent Kit (Thermo Fisher, Waltham, MA, USA) and a TBGreen Premix Ex Taq^TM^ II Kit (Takara Bio, Inc., Tokyo, Japan) were used to perform reverse transcription and real-time PCR, respectively. The primer sequences are shown in Table S1. The levels of mRNA expression in the HSP genes were analyzed using the CFX96 Real-time PCR System (Bio-Rad, Hercules, CA, USA) and were calculated using the Livak method (2^−ΔΔCT^). *Gapdh* was used as a housekeeping gene for normalization.

### 2.7. Chromatin Immunoprecipitation (ChIP) Assay

A ChIP assay was performed using the kit listed in a previous study [26]. Histone–DNA complexes in liver homogenates were immunoprecipitated using the antibodies against IgG, H3 and H3K18ac. Among them, IgG and H3 served as negative and positive controls, respectively. Quantitative real-time PCR was used to measure the gene copy number of the purified DNA eluted from the complexes. Six pairs of primers were designed for each HSP gene for ChIP-qPCR to determine the enrichment of H3K18ac in the promoter regions (−1000 bp upstream and +1000 bp downstream to the transcription start site) (Table S2).

### 2.8. Statistical Analysis

Statistical analysis was performed using SPSS version 21.0 (SPSS Inc., Chicago, IL, USA). Differences between the two groups were analyzed using a t-test (normal variables) or an independent sample non-parametric test (non-normal variables). Spearman correlation analysis was used to analyze the association between the liver arsenic and other normal variables, and the correlations between the normal variables were assessed using Pearson correlation analysis. Differences were considered statistically significant when *p* < 0.05.

## 3. Results

### 3.1. Arsenic-Exposure-Induced Liver Injury in Rat

**Figure 1:**
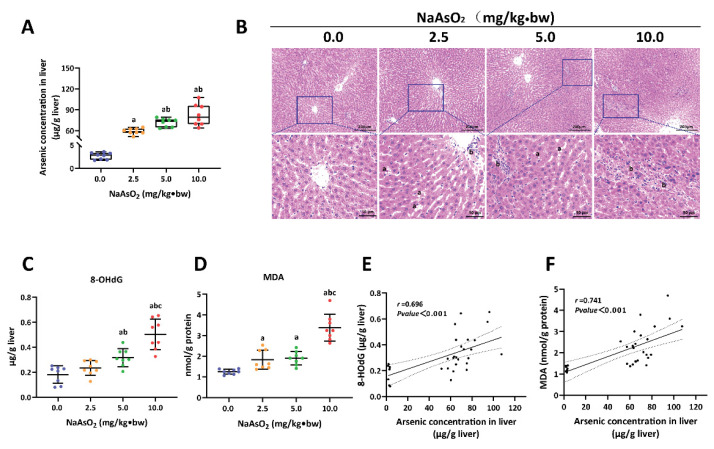
The liver injury of rats induced by NaAsO_2._ (**A**) Arsenic concentration, (**B**) pathological changes, and (**C**,**D**) the levels of 8-OHdG and MDA in the liver of rats with different doses of NaAsO_2_ treatment. (**E**,**F**) The correlation between the arsenic concentration in the liver (LA) and the levels of 8-OHdG and MDA. In (**A**,**C**,**D**), a, b and c represent *p* < 0.05 compared with the group of 0.0, 2.5 and 5.0 mg/kg·bw NaAsO_2_, respectively. In (**B**), a and b represent steatosis and inflammatory cell infiltration, respectively.

The rat model of liver injury induced by arsenic was constructed using 0, 2.5, 5.0 and 10.0 mg/kg of NaAsO_2_ treatment for 16 weeks. As previously mentioned, the arsenic-exposed rats exhibited varying degrees of poisoning symptoms, such as fluffy hair, slowed response and decreased activity. These symptoms were exacerbated in the 10.0 mg/kg of NaAsO_2_ treatment group. From the beginning of the twelfth week, the body weight of the arsenic-exposed rats tended to decrease compared with the control group (The data are detailed in the citation) [8]. The arsenic concentration in the liver tissue (LA) was measured to indicate internal exposure. As shown in Figure 1A, the levels of LA rose with the increase in the NaAsO_2_ treatment dose. Then, the histopathological characteristics of the liver tissue were assessed via HE staining. Compared with the control group, different degrees of hepatic sinusoidal congestion, steatosis and inflammatory cell infiltration were observed in the arsenic groups (Figure 1B). In addition, our previous study showed that arsenic exposure decreased the levels of albumin (ALB), albumin/globulin (A/G) and cholinesterase (CHE), and elevated the level of alanine aminotransferase (ALT) in serum (The data are detailed in the citation) [8].

To further explore whether arsenic exposure induces hepatic oxidative damage in rats, we measured the levels of 8-OhdG and MDA in the liver, which are reliable markers of DNA and lipid peroxidation, respectively. The results showed that the trends of increased 8-OhdG and MDA corresponded to the raised dose of NaAsO_2_ treatment (Figure 1C,D). Moreover, a significant positive correlation between the levels of 8-OhdG (*r* = 0.696), MDA (*r* = 0.741) and LA was found in the Spearman analysis (Figure 1E,F). These results suggest that subchronic exposure to arsenic can induce oxidative damage and subsequent histopathological lesions in the liver of rats.

### 3.2. H3K18ac Was Associated with Hepatic Oxidative Damage Induced by Arsenic

**Figure 2:**
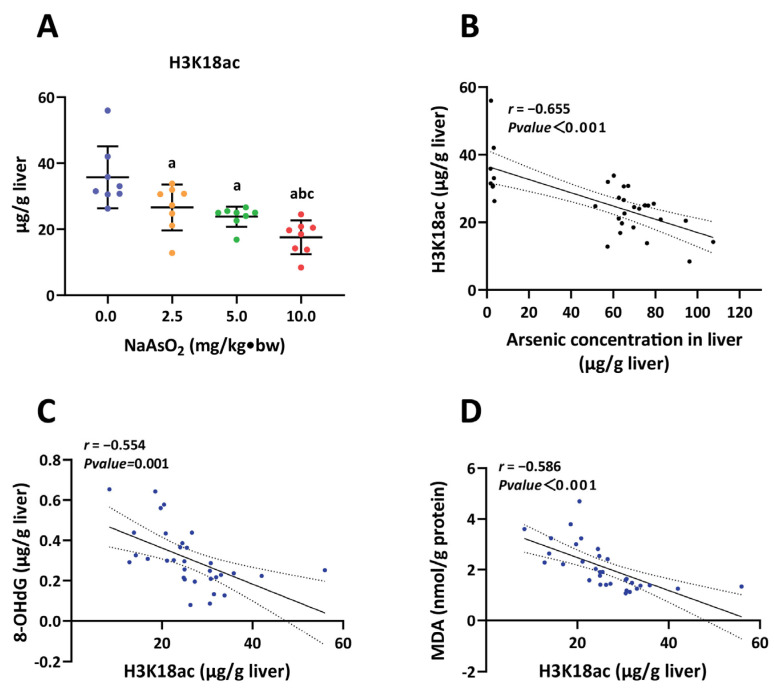
H3K18ac in response to the hepatic oxidative damage of rats induced by NaAsO_2_. (**A**) The levels of H3K18ac in the liver of rats with different doses of NaAsO_2_ treatment. a, b and c represent *p* < 0.05 compared with the group of 0.0, 2.5 and 5.0 mg/kg·bw NaAsO_2_, respectively. (**B**) The correlations between LA and the level of H3K18ac. (**C**,**D**) The correlation between H3K18ac and the levels of 8-OHdG and MDA.

Next, we measured the level of H3K18ac in the rat liver using ELISA to assess whether the modification of H3K18ac is associated with arsenic-induced hepatic oxidative damage. As shown in Figure 2A, the levels of H3K18ac in the arsenic groups decreased remarkably compared to the controls, and the trend of reduced H3K18ac responded to the NaAsO_2_ treatment in a dose-dependent manner. Consistently, the reduction in H3K18ac was associated with increased levels of LA (*r* = −0.655) (Figure 2B). Furthermore, we performed Pearson correlation analysis to explore the relationship between H3K18ac and hepatic oxidative damage, and found that the decreased H3K18ac levels were accompanied by the enhanced 8-OHdG (*r*= −0.554) and MDA (*r*= −0.586) levels (Figure 2C,D). Taken together, these results indicate that H3K18ac is potentially a modifiable epigenetic marker in the liver in response to arsenic exposure, and the aberrant H3K18ac may be involved in mediating hepatic oxidative damage induced by arsenic.

### 3.3. H3K18ac Might Be Involved in Arsenic-Induced Hepatic Oxidative Damage by Regulating Expression of Heat Shock Proteins

**Figure 3:**
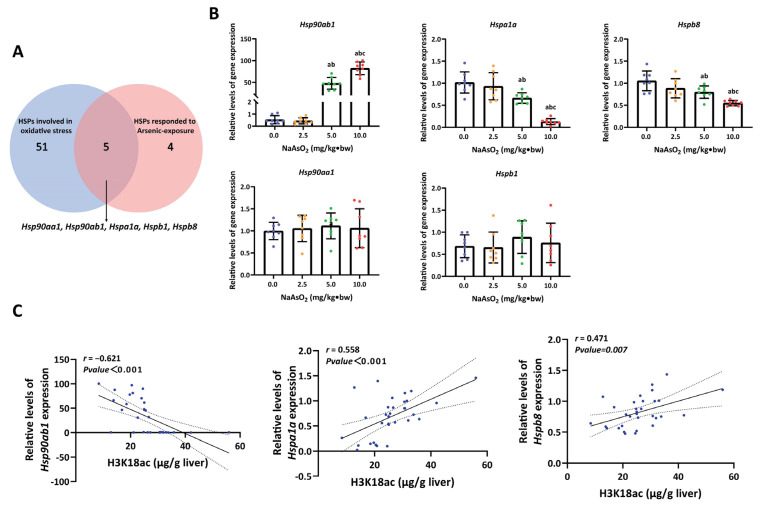
The association between HSP gene expression and H3K18ac in the liver of rats upon NaAsO_2_ treatment. (**A**) Two subsets, for HSP genes either involved in oxidative stress or responding to arsenic exposure, were obtained from databases. Five representative HSP genes, including *Hsp90aa1, Hsp90ab1, Hspa1a, Hspb1* and *Hspb8*, which might be involved in hepatic oxidative damage induced by arsenic, were identified from two subsets via Venn analysis. (**B**) Dose-dependent induction of the expression of five representative HSP genes by NaAsO_2_. a, b, c represents *p* < 0.05 compared with the group of 0.0, 2.5 and 5.0 mg/kg·bw NaAsO_2_, respectively. (**C**) Aberrant expression of *Hsp90ab1, Hspa1a* and *Hspb8* corresponded to the change in H3K18ac modification.

Heat shock proteins are important scavengers of cellular oxidative damage. In this study, HSPs were targeted to further define how H3K18ac responds to hepatic oxidative damage. Firstly, we obtained 9 HSP genes that respond to arsenic exposure by analyzing gene expression profiles (GSE19662) from the GEO database (Table S3), and 56 HSP genes related to oxidative stress were captured from the GeneCards database (Table S4). Five overlapping genes that might be involved in arsenic-induced oxidative damage were identified via Venn analysis, including *Hsp90ab1*, *Hspa1a*, *Hspb8*, *Hsp90aa1* and *Hspb1* (Figure 3A). Next, we examined the expressions of these five HSP genes in rat liver. As a result, three genes, including *Hsp90ab1, Hspa1a* and *Hspb8*, responded to NaAsO_2_ treatment. Among them, the level of *Hsp90ab1* expression increased, while the expression of *Hspa1a* and *Hspb8*a remarkably decreased in a dose-dependent manner (Figure 3B). Similar results were also obtained in a Spearman correlation analysis between LA and the expression of HSPs (Table S5). Accordingly, the trends observed in the increased expression of *Hsp90ab1* and the reduced expression of *Hspa1a* and *Hspb8* corresponded to the decreasing H3K18ac modification (Figure 3C). Based on the function of H3K18ac in transcriptional activation, we speculate that *Hspa1a* and *Hspb8*a may be the representative HSP genes involved in hepatic oxidative damage mediated by H3K18ac modification.

**Figure 4:**
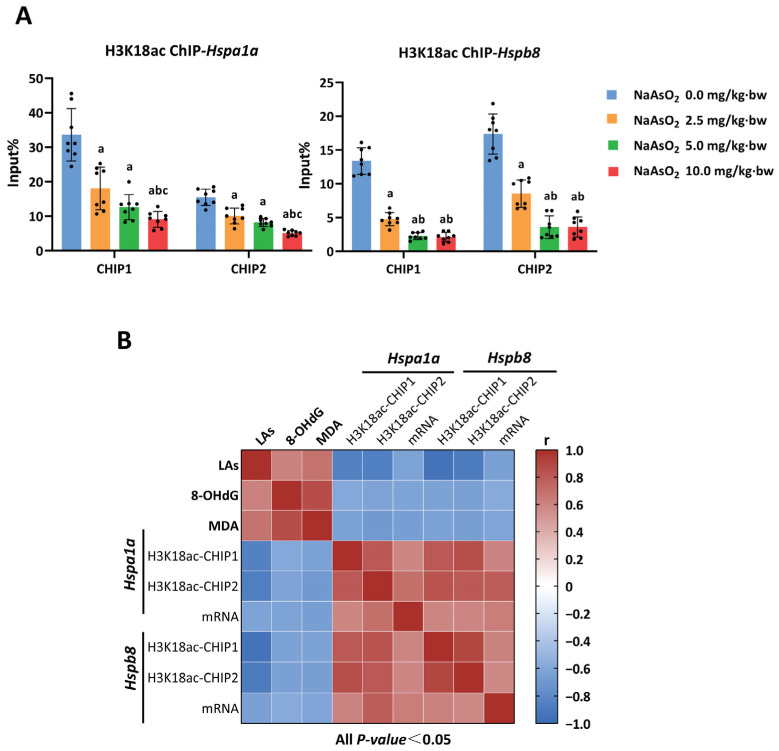
The enrichment of H3K18ac in HSP genes responded to LA, gene expression, and hepatic oxidative damage upon NaAsO_2_ treatment. (**A**) The amount of enrichment of the H3K18ac modification in the promoters of *Hspa1a* and *Hspb8* was measured using ChIP-qPCR. “Input%” means the percent of chromatin precipitated by H3K18ac antibodies relative to the total input of sheared chromatin. a, b and c represent *p* < 0.05 compared with the group of 0.0, 2.5 and 5.0 mg/kg·bw NaAsO_2_, respectively. (**B**) Matrix correlation analysis was used to comprehensively demonstrate the relationships between LA, the enrichment of H3K18ac, the expression of *Hspa1a* and *Hspb8*, and hepatic oxidative damage (8-OHdG and MDA). Blue squares represent negative correlations, whereas red squares are positive, with darker colors indicating more significant correlations.

Next, a chromatin immunoprecipitation-quantitative PCR (ChIP-qPCR) assay was performed to examine whether H3K18ac plays a role in transcriptional regulation via a direct interaction with the promoter regions of the *Hsp90ab1, Hspa1a* and *Hspb8* genes. The quality controls of ChIP-qPCR are shown in Figure S1. The enrichment levels of the negative control IgG in the promoter regions were less than 1/10 of the enrichment of the positive control H3 and H3K18ac, and the enrichment levels of the positive control H3 in the promoter regions were 2%–8% input. The enrichments of H3K18ac were measured in six DNA fragments for each gene. As shown in Figure 4A, we found that the enrichment of H3K18ac on two fragments of the promoter regions of *Hspa1a* and *Hspb8* genes created a decreased response to NaAsO_2_ treatment in a dose-dependent manner. However, no obvious changes were observed in other fragments of these two genes or in all fragments of *the Hsp90ab1* genes (Figure S2). Remarkably, matrix correlation analysis was used to comprehensively demonstrate the relationships between arsenic load, the enrichment of H3K18ac, the expression of HSP genes, and hepatic oxidative damage. As shown in Figure 4B, the enrichment of H3K18ac in the *Hspa1a* and *Hspb8* genes tended to be gradually reduced following accumulated arsenic load in the liver, which corresponded to a reduction in the gene expressions and the aggravation of hepatic oxidative damage. Taken together, these observations suggest that the transcriptional inhibition of the *Hspa1a* and *Hspb8* genes was regulated by a reduction in H3K18ac enrichment, which plays a critical role in arsenic-induced hepatic oxidative damage.

### 3.4. R. roxburghii Could Alleviate Arsenic-Induced Liver Injury by Declining Oxidative Damage

**Figure 5:**
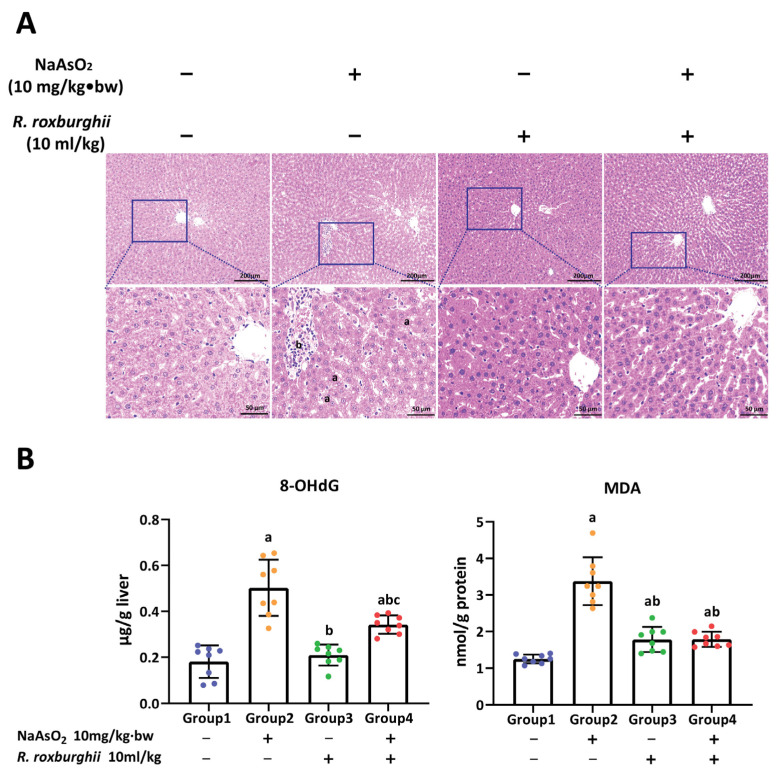
The antagonistic effect of *R. roxburghii* juice on arsenic-induced liver injury. (**A**) Pathological changes and (**B**) the levels of 8-OHdG and MDA in the liver of rats in controls, arsenic group (10.0 mg/kg·bw NaAsO_2_), single *R. roxburghii* juice group (10 mL/kg *R. roxburghii* juice) and *R. roxburghii* juice antagonist group (10.0 mg/kg·bw NaAsO_2_ + 10 mL/kg *R. roxburghii* juice). In (**A**), a and b represent steatosis and inflammatory cell infiltration, respectively. In (**B**), a, b and c represent *p* < 0.05 compared with the controls, arsenic group and single *R. roxburghii* juice group, respectively.

To identify the antagonistic effect of *R. roxburghii* on arsenic-induced liver injury in rats, a model of *R. roxburghii* juice intervention was constructed, including the single *R. roxburghii* juice group (10 mL/kg *R. roxburghii* juice) and the *R. roxburghii* juice antagonist group (10.0 NaAsO_2_ mg/kg + 10 mL/kg *R. roxburghii* juice) around the same time. As shown in Figure 5A, steatosis and inflammatory cell infiltration were observed in arsenic groups, while only slight hepatic sinusoidal dilatation and congestion were found in the *R. roxburghii* juice antagonist group. No obvious pathological changes were observed in the single *R. roxburghii* juice group and the control group. Although the levels of 8-OHdG and MDA in the *R. roxburghii* juice antagonist group were higher than those in the control group, a remarkable reduction was observed compared to the arsenic group (Figure 5B). These observations suggest that *R. roxburghii* juice could alleviate arsenic-induced hepatic oxidative damage and subsequent histopathological lesions.

### 3.5. The Antioxidative Effect of R. Roxburghii Might Be Involved in Antagonizing Arsenic-induced Inhibition of the H3K18ac–HSPs Axis

**Figure 6:**
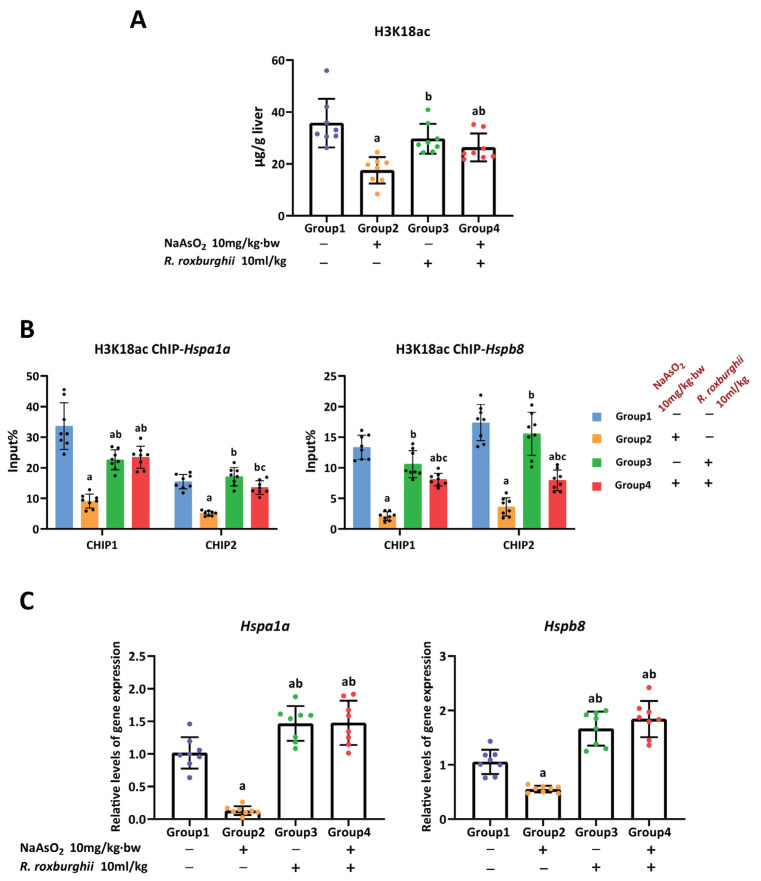
*R. roxburghii* juice alleviated the inhibition of the H3K18ac–HSPs axis induced by arsenic. (**A**) Modification of H3K18ac, (**B**) enrichment of H3K18ac in promoters of *Hspa1a* and *Hspb8* genes and (**C**) expression of *Hspa1a and Hspb8* genes in the liver of rats in controls, arsenic group (10.0 mg/kg·bw NaAsO_2_), single *R. roxburghii* juice group (10 mL/kg *R. roxburghii* juice) and *R. roxburghii* juice antagonist group (10.0 mg/kg·bw NaAsO_2_ + 10 mL/kg *R. roxburghii* juice). a, b and c represent *p* < 0.05 compared with controls, arsenic group and single *R. roxburghii* juice group, respectively.

The results listed above demonstrate that a reduction in H3K18ac enrichment in the promoters of the *Hspa1a* and *Hspb8* genes could inhibit the genes’ expression, which is involved in enhanced hepatic oxidative damage induced by arsenic. We attempt to identify whether the H3K18ac–HSPs axis plays a role in the protective effect of *R. roxburghii* juice on arsenic-induced hepatic oxidative damage. The results showed that *R. roxburghii* juice could mitigate the arsenic-induced reduction in H3K18ac modification in the liver (Figure 6A). Consistently, the results of the ChIP-qPCR revealed that the enrichment of H3K18ac in the promoters of the *Hspa1a* and *Hspb8* genes was restored in the *R. roxburghii* juice antagonist group, compared with the arsenic group (Figure 6B). Accordingly, the trends observed in the increased expression of the *Hspa1a* and *Hspb8* genes corresponded to the restored enrichment of H3K18ac (Figure 6C). The quality control of the ChIP-qPCR is shown in Figure S3. Furthermore, we measured the effect of *R. roxburghii* juice on the expression of three non-targeted heat shock protein genes (*Hsp90ab1*, *Hsp90aa1*, *Hspb1*) and found that *R. roxburghii* juice did not affect the expression of *Hsp90aa1* and *Hspb1;* however, it was able to increase the *Hsp90ab1* expression, while no apparent change in H3K18ac enrichment was observed in the promoter region of *Hsp90ab1*, suggesting that the increased expression of *Hsp90ab1* after *R. roxburghii* juice treatment might not be the result of H3K18ac regulation (Figure S4). Taken together, these results confirmed the notion that H3K18ac–HSPs play a critical role in the response to the hepatic oxidative damage induced by arsenic and revealed that *R. roxburghii* juice could antagonize hepatic oxidative damage by alleviating the burden of arsenic exposure on the H3K18ac–HSPs axis.

## 4. Discussion

Liver injury and its subsequent irreversible progression are some of the major health threats of environmental arsenic exposure. Oxidative damage is a critical event in triggering arsenic-induced liver diseases. In this study, the rat model of liver injury induced by arsenic was used to reveal that H3K18ac might be a critical epigenetic regulator of arsenic-induced hepatic oxidative damage. Furthermore, a novel mechanism was uncovered, showing that a reduction in H3K18ac was involved in arsenic-induced hepatic oxidative damage through the transcriptional inhibition of *Hspa1a* and *Hspb8*. Notably, based on the H3K18ac–HSPs axis-dependent antioxidative pathway, we identified the active role and mechanism of *R. roxburghii* juice in antagonizing arsenic-induced liver injury.

The role of H3K18ac in hepatic diseases is gradually being recognized. An increased level of H3K18ac was found to promote acute liver injury via the activation of inflammation and macrophage polarization, and the inhibitor of histone acetyltransferase p300/CBP was demonstrated to alleviate acute liver injury by correcting this epigenetic distortion [34]. In addition, increased H3K18ac has been observed to respond to Hepatitis B Virus (HBV) infection [35]. Unlike the abnormal elevation of H3K18ac in previous studies, we found that a reduction in H3K18ac is the main aberrant mode in response to hepatic oxidative damage induced by sub-chronic arsenic exposure. These results suggest that the disturbance mode of H3K18ac may be complex in response to different types of liver injury. The observation in this study will require further experimental study to better inform regarding the overall role of H3K18ac as a determinant of oxidative stress. Notably, the role of H3K18ac in the adverse outcomes induced by environmental pollutants also deserves attention. This study revealed that H3K18ac could respond to arsenic exposure and participate in arsenic-induced liver injury. Similarly, a reduction in H3K18ac was also found to be involved in hexavalent chromium tumorigenicity [36], while it has been found that environmental pollutants, such as perfluorooctane sulfonate [37] and particulate matters [38], perturb the transcription of related genes by increasing H3K18ac. Although H3K18ac responds differently to different pollutant exposures, distorted H3K18ac might be a critical epigenetic regulator of pollutant toxicity. Therefore, the role of H3K18ac as a biomarker for the risk assessment of environmental pollutants is also worth looking into.

The value of epigenetic modifications as a target of precision therapy seems to be more desirable because of their reversibility and sensitivity to disease progression and prognosis [39,40]. Epigenetic modification has been demonstrated to play an important role in the transcriptional regulation of HSP40, HSP60, HSP70, HSP90 and HSP110 [41]. The expression inhibition of *Hspa1a* and *Hspb8* was found to be regulated by DNA hypermethylation [42,43]. Treatment with the DNA methyltransferase inhibitor 5-Aza-2′-deoxycytidine (5-AZA) could improve cellular disorders by targeting the correction of the expression of these two genes [44,45]. The effect of broad-spectrum histone deacetylase (HDAC) on the expression of *Hspa1a* and *Hspb8* indirectly proved that histone acetylation is another epigenetic regulator of these two genes [46,47]. However, the regulation of gene expression by either 5-AZA or HDAC lacks specificity. Fortunately, more and more site-specific modification enzymes of histone have been uncovered. For example, SETD2 is responsible for catalyzing the methylation of H3K36 [48,49]. These findings mean that aberrant histone modification in a specific site can be targeted more precisely to correct the abnormal gene expression regulated by the site. Our results revealed that the enrichment levels of H3K18ac in the promoter regions of *Hspa1a* and *Hspb8* affected the transcriptional capacity of these two genes, which provides a precise epigenetic target for specific interventions that target these two heat shock proteins.

Antioxidant activity is one of the main effects of *R. roxburghii* [32]. The antioxidant mechanism of *R. roxburghii* is under intense investigation in order to promote the application of *R. roxburghii* in disease therapy and health care. Emerging evidence reveals that the Nrf2-mediated antioxidant pathway [50], the PPARα-mediated mitochondrial homeostasis pathway [51] and the activation of oxidative-stress-responsive genes [52] might be the main antioxidant mechanism of *R. roxburghii*. For the first time, this study provides evidence of the epigenetic mechanism of the antioxidant effects of *R. roxburghii* juice. We demonstrate that *R. roxburghii* juice could antagonize arsenic-induced oxidative damage by regulating the H3K18ac-dependent transcriptional activation of HSP genes. Although we could not explain the mechanism of how *R. roxburghii* influences histone modifications based on the existing literature, the emerging evidence suggests that natural antioxidant components might affect the expression or activity of methylation- and acetylation-modifying enzymes to regulate the level of DNA methylation and histone modifications [53,54]. This evidence provides the clue for our follow-up work.

There are some limitations to this study. Firstly, while this study revealed the role of the H3K18ac-dependent transcriptional regulation of HSP genes in arsenic-induced rat liver toxicity and associated oxidative damage, additional follow-up experiments are required to further explore the biological significance of the hypothesis as a modulator of chemically induced liver injury, as well as human liver diseases. In addition, future research should further explore the mechanisms of arsenic toxicity in *R. roxburghii* juice antagonism, based on the consideration of the effects of *R. roxburghii* juice on the systemic absorption and excretion of arsenic.

## 5. Conclusions

**Figure 7:**
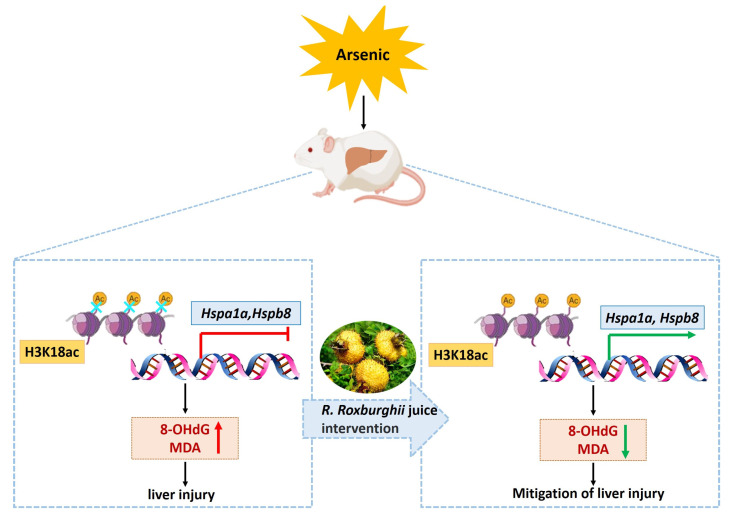
Reduction in H3K18ac is involved in arsenic-induced hepatic oxidative damage via the modulation of the transcriptional inhibition of HSP genes. *Rosa roxburghii Tratt* juice antagonized arsenic-induced hepatic oxidative damage, thereby alleviating histopathological lesions by recovering the transcriptional activation of the *Hspa1a* and *Hspb8* genes modulated by H3K18ac.

This study reveals that a reduction in H3K18ac is involved in arsenic-induced hepatic oxidative damage by modulating the transcriptional inhibition of HSP genes. *R. roxburghii* juice could act against arsenic-induced hepatic oxidative damage, thereby alleviating histopathological lesions by rescuing the H3K18ac-dependent transcriptional activation of HSP genes (Figure 7). These findings uncover a novel epigenetic mechanism of *R. roxburghii* juice in antagonizing arsenic-induced liver injury and provide useful evidence for the translational application of *R. roxburghii* juice in the prevention of liver injury.

## Data Availability

The data presented in this study are available upon request from the corresponding author.

## Supplementary Materials

The following supporting information can be downloaded at: https://www.mdpi.com/article/10.3390/toxics11060503/s1, Table S1 The primers for qRT-PCR. Table S2 The primers for ChIP-qPCR. Table S3 HSPs genes response to arsenic-exposure obtained from GSE19662. Table S4 HSPs genes involved in oxidative stress from GeneCard. Table S5 Correlation between the concentration of liver arsenic and the mRNA levels of heat shock proteins in rat liver. Fig. S1 The enrichment of H3 (A) and IgG (B) in promoters of *Hsp90ab1*, *Hspa1a* and *Hspb1* genes in liver of rats with different doses of NaAsO_2_ treatment. Fig. S2 The enrichment of H3K18ac in promoters’ fragments of *Hspa1a* (A), *Hspb8* (B) and *Hsp90ab1* (C) genes in liver of rats with different doses of NaAsO_2_ treatment. Fig. S3 The enrichment of H3, IgG and H3K18ac in promoters of *Hspa1a* (A) and *Hspb1* (B) genes in liver of rats in controls, arsenic group, single *R. roxburghii* juice group and *R. roxburghii* juice antagonist group. Fig. S4 Expression of *Hsp90aa1*, *Hspb1* and *Hsp90ab1* (A) and the enrichment of H3K18ac (B), H3 and IgG (C) in the gene promoters of rats’ liver in controls, arsenic group, single *R. roxburghii* juice group and *R. roxburghii* juice antagonist group.
