# Supplementary material for: Inhibition of Histone H3K18 Acetylation-Dependent Antioxidant Pathways Involved in Arsenic-Induced Liver Injury in Rats and the Protective Effect of Rosa roxburghii Tratt Juice
Source: Toxics. 2023 Jun 3;11(6):503. doi: 10.3390/toxics11060503 (PMC10305127; doi:10.3390/toxics11060503)
Supplement: Supplementary file 1 [file toxics-11-00503-s001.zip › toxics-2378335-supplementary.pdf]

Table S1 The primers for qRT-PCR.

Genes	Products length (bp)	Primer (5'-3')	
Hsp90aa1	208	Forward	AGTTGTATGTTCGCAGAGT
		Reverse	TTATCTTCAGCCAGTTCAGT
Hsp90ab1	290	Forward	TCCTTCGCTACCATACCT
		Reverse	CTTCTCTTCCTCATCCTCTG
Hspa1a	261	Forward	CACCATCACCAACGACAAGG
		Reverse	TCAGCCAGCGTGTTAGAGTC
Hspb1	81	Forward	ACTGGCAAGCACGAAGAAAGG
		Reverse	ACCTGGAGGGAGCGTGTATTT
Hspb8	149	Forward	CTGCTGGACGATGGCTTTG
		Reverse	AACCTGGCTGTGGCTGTAG
**GAPDH**	122	Forward	AAGTTCAACGGCACAGTCAAG
		Reverse	ACATACTCAGCACCAGCATCA

Table S2 The primers for ChIP-qPCR.

Genes	Location (TSS)		Primer (5'-3')
Hspa1a			
CHIP1	+58～+201	Forward	GTGTTCCAGCACGGCAAGGT
		Reverse	CACGGTGTTCTGCGGGTTCA
CHIP2	+280～+394	Forward	GTGGTGAACGACGGCGACAA
		Reverse	CGGCGATCTCCTTCATCTTGGT
CHIP3	+639～+769	Forward	CGACGGCATCTTCGAGGTGAA
		Reverse	TGTTCTGGCTGATGTCCTTCTTGT
CHIP4	-194～-86	Forward	GCTTCACATACAGAGACCGCTACC
		Reverse	GGCTTCGCTGGACAGATTGGAT
CHIP5	-336～-148	Forward	AGACCCGAAACTGCTGGAAGATTC
		Reverse	GCTCCGCTTCTCTGCTTCTCTG
CHIP6	-861～-723	Forward	ACTGTGCGAGTCTGCGAACC
		Reverse	CCTGCTGGTGCTGCTGTTGA
Hspb8			
CHIP1	-169 ～ -69	Forward	TCTGGGCAGCGGATGTTTGG
		Reverse	AGCAAGGTGATGTGGCTTGAGAT
CHIP2	+354 ～ +472	Forward	CGTGGAAGTGTCAGGTGAGTGA
		Reverse	CGCATAAGAGCACAGGCATGG
CHIP3	+475 ～ +635	Forward	TGCTCCCAGGTTCATCCCAGAA
		Reverse	TCCATCACATCCACGCTCATTCC
CHIP4	+667 ～ +794	Forward	GGGTGGTAATGTCAGCAGTAGGG
		Reverse	ACGCACCAGCCATGTCCTCT
CHIP5	-594 ～ -488	Forward	AGGAGGAGATCGTGCCTGAGAG
		Reverse	TCCCTGAGAATCCGCTGAGAGAA
CHIP6	-927 ～ -744	Forward	CTGCCTGACAACGTCCTGCTT
		Reverse	GTTCTGTCTTGCCTCCCACTCTG
Hsp90ab1			
CHIP1	-1329～-1174	Forward	TGAAGGACAGGCAAGGCATGG
		Reverse	GAGAGGAGGAGGTCACGGTCTT
CHIP2	-911～-776	Forward	CGCTCCACACCTCCACAGTAAT
		Reverse	TCGCCTTTCGGCTGGTTCTTT
CHIP3	-449～-232	Forward	CGAATTGGTCACCTCCGTTCTCTT
		Reverse	GGATGTATCTGCGGCGAATACCC
CHIP4	+29～+183	Forward	TCGGCTTTCTCGTCAAGGTAAGG
		Reverse	CCACTGGAGACATTCTAGGCACAT
CHIP5	+285～+427	Forward	ACAGTTCAGCGTTCATGGTGGA
		Reverse	AGAAAGAGCAGCCTCCCGTAAAC
CHIP6	+391～+508	Forward	ATGACTCAGCAAGACGAGGTTGG
		Reverse	GGACAAACGCAGTGGGTTCAAG

Table S3 HSPs genes response to arsenic-exposure obtained from GSE19662

Gene symbol	Log Fold Change	Average Expression	*t*	*P.Value*
Hsp90aa1	0.73003883	1.149383597	2.307498556	0.035002895
Hsp90ab1	0.72385935	0.666948773	2.585104709	0.020150031
Hspa1a	3.40052823	3.737665442	5.050132814	0.000125106
Hspb1	1.808126168	1.52577845	2.928125733	0.009998273
Hspb8	1.155033808	0.720940701	2.319342477	0.034199765
Hsph1	1.911481326	1.56739521	3.151599961	0.006288684
DNAJB1	1.378487932	1.233799915	2.788172678	0.013333562
DNAJA4	1.231813563	0.843895513	3.06176331	0.007580871
DNAJC5	0.630824091	0.771079719	2.137948336	0.048605355

Table S4 HSPs genes involved in oxidative stress from GeneCard

Gene Symbol	Description
HSPA5	Heat Shock Protein Family A (Hsp70) Member 5
HSP90AA1	Heat Shock Protein 90 Alpha Family Class A Member 1
HSPB1	Heat Shock Protein Family B (Small) Member 1
HSPA4	Heat Shock Protein Family A (Hsp70) Member 4
HSPA1A	Heat Shock Protein Family A (Hsp70) Member 1A
HSPA8	Heat Shock Protein Family A (Hsp70) Member 8
HSPA9	Heat Shock Protein Family A (Hsp70) Member 9
HSPD1	Heat Shock Protein Family D (Hsp60) Member 1
HSP90B1	Heat Shock Protein 90 Beta Family Member 1
HSPA1B	Heat Shock Protein Family A (Hsp70) Member 1B
HSP90AB1	Heat Shock Protein 90 Alpha Family Class B Member 1
HSPA14	Heat Shock Protein Family A (Hsp70) Member 14
HSPA13	Heat Shock Protein Family A (Hsp70) Member 13
DNAJC3	DnaJ Heat Shock Protein Family (Hsp40) Member C3
HSPA6	Heat Shock Protein Family A (Hsp70) Member 6
DNAJB1	DnaJ Heat Shock Protein Family (Hsp40) Member B1
HSPB2	Heat Shock Protein Family B (Small) Member 2
HSPB8	Heat Shock Protein Family B (Small) Member 8
HSPA2	Heat Shock Protein Family A (Hsp70) Member 2
DNAJA1	DnaJ Heat Shock Protein Family (Hsp40) Member A1
HSPE1	Heat Shock Protein Family E (Hsp10) Member 1
HSPA1L	Heat Shock Protein Family A (Hsp70) Member 1 Like
DNAJC10	DnaJ Heat Shock Protein Family (Hsp40) Member C10
HSPB6	Heat Shock Protein Family B (Small) Member 6
DNAJB9	DnaJ Heat Shock Protein Family (Hsp40) Member B9
HSPH1	Heat Shock Protein Family H (Hsp110) Member 1
DNAJB11	DnaJ Heat Shock Protein Family (Hsp40) Member B11
HSPA12A	Heat Shock Protein Family A (Hsp70) Member 12A
HSBP1	Heat Shock Factor Binding Protein 1
HSPA4L	Heat Shock Protein Family A (Hsp70) Member 4 Like
HSPA12B	Heat Shock Protein Family A (Hsp70) Member 12B
DNAJC12	DnaJ Heat Shock Protein Family (Hsp40) Member C12
DNAJB2	DnaJ Heat Shock Protein Family (Hsp40) Member B2
DNAJB6	DnaJ Heat Shock Protein Family (Hsp40) Member B6
DNAJC30	DnaJ Heat Shock Protein Family (Hsp40) Member C30

Continue Table S4 HSPs genes involved in oxidative stress from GeneCard

Gene Symbol	Description
DNAJA2	DnaJ Heat Shock Protein Family (Hsp40) Member A2
DNAJB4	DnaJ Heat Shock Protein Family (Hsp40) Member B4
DNAJC7	DnaJ Heat Shock Protein Family (Hsp40) Member C7
DNAJC19	DnaJ Heat Shock Protein Family (Hsp40) Member C19
DNAJA3	DnaJ Heat Shock Protein Family (Hsp40) Member A3
DNAJB1P1	DnaJ Heat Shock Protein Family (Hsp40) Member B1 Pseudogene 1
DNAJC2	DnaJ Heat Shock Protein Family (Hsp40) Member C2
DNAJB5	DnaJ Heat Shock Protein Family (Hsp40) Member B5
DNAJC17	DnaJ Heat Shock Protein Family (Hsp40) Member C17
HSPB7	Heat Shock Protein Family B (Small) Member 7
DNAJB12	DnaJ Heat Shock Protein Family (Hsp40) Member B12
HSPA7	Heat Shock Protein Family A (Hsp70) Member 7 (Pseudogene)
DNAJC15	DnaJ Heat Shock Protein Family (Hsp40) Member C15
DNAJC11	DnaJ Heat Shock Protein Family (Hsp40) Member C11
DNAJC6	DnaJ Heat Shock Protein Family (Hsp40) Member C6
DNAJB3	DnaJ Heat Shock Protein Family (Hsp40) Member B3
DNAJA4	DnaJ Heat Shock Protein Family (Hsp40) Member A4
DNAJC9	DnaJ Heat Shock Protein Family (Hsp40) Member C9
DNAJC1	DnaJ Heat Shock Protein Family (Hsp40) Member C1
DNAJC16	DnaJ Heat Shock Protein Family (Hsp40) Member C16
DNAJC24	DnaJ Heat Shock Protein Family (Hsp40) Member C24
DNAJC5	DnaJ Heat Shock Protein Family (Hsp40) Member C5
DNAJC4	DnaJ Heat Shock Protein Family (Hsp40) Member C4
HSPD1P9	Heat Shock Protein Family D (Hsp60) Member 1 Pseudogene 9

Table S5 Correlation between the concentration of liver arsenic and the mRNA levels of heat shock proteins in rat liver

mRNA levels (fold change)	n	liver arsenic (μg/g liver)	
		**Correlation coefficient of spearman**	***P-value***
Hsp90ab1	32	0.759	＜0.001
Hspa1a	32	−0.687	＜0.001
Hspb8	32	−0.700	＜0.001
Hsp90aa1	32	0.070	0.703
Hspb1	32	0.019	0.918

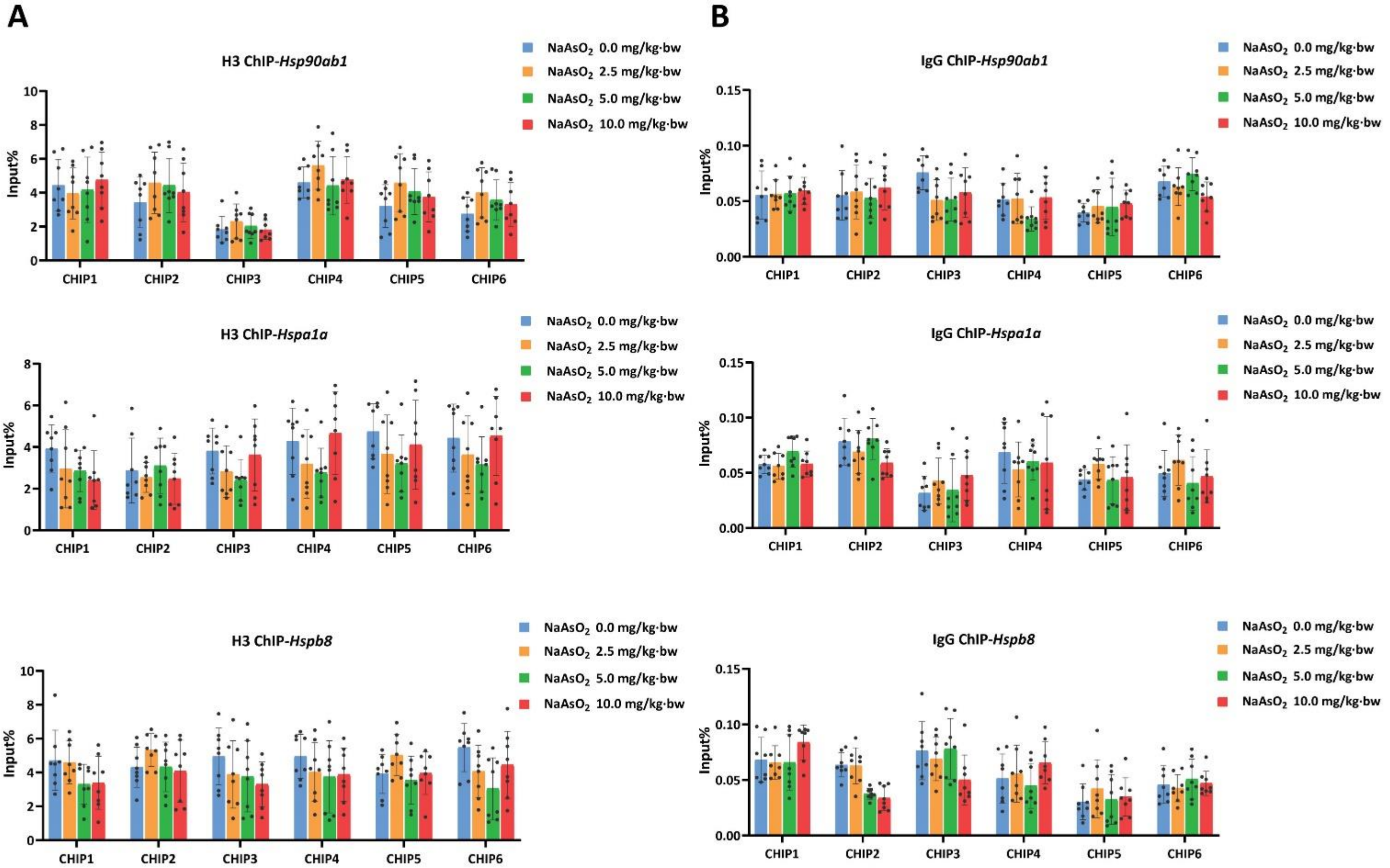

Figure S1 The enrichment of H3 (A) and IgG (B) in promoters of *Hsp90ab1*, *Hspa1a* and *Hspb1* genes in liver of rats with different doses of $NaAsO_2$ treatment.

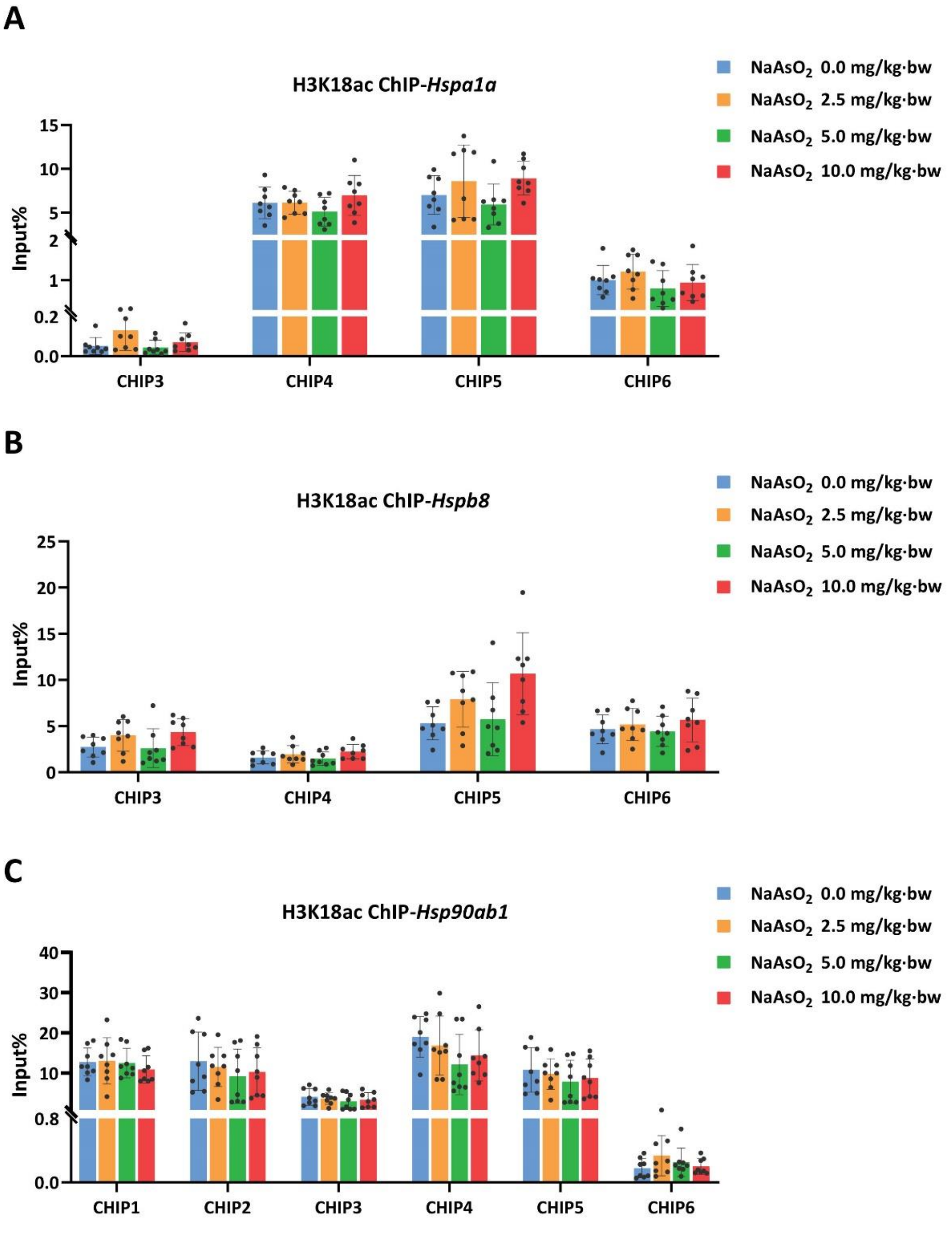

Figure S2 The enrichment of H3K18ac in promoters' fragments of *Hspa1a* (A), *Hspb8* (B) and *Hsp90ab1* (C) genes in liver of rats with different doses of $NaAsO_2$ treatment.

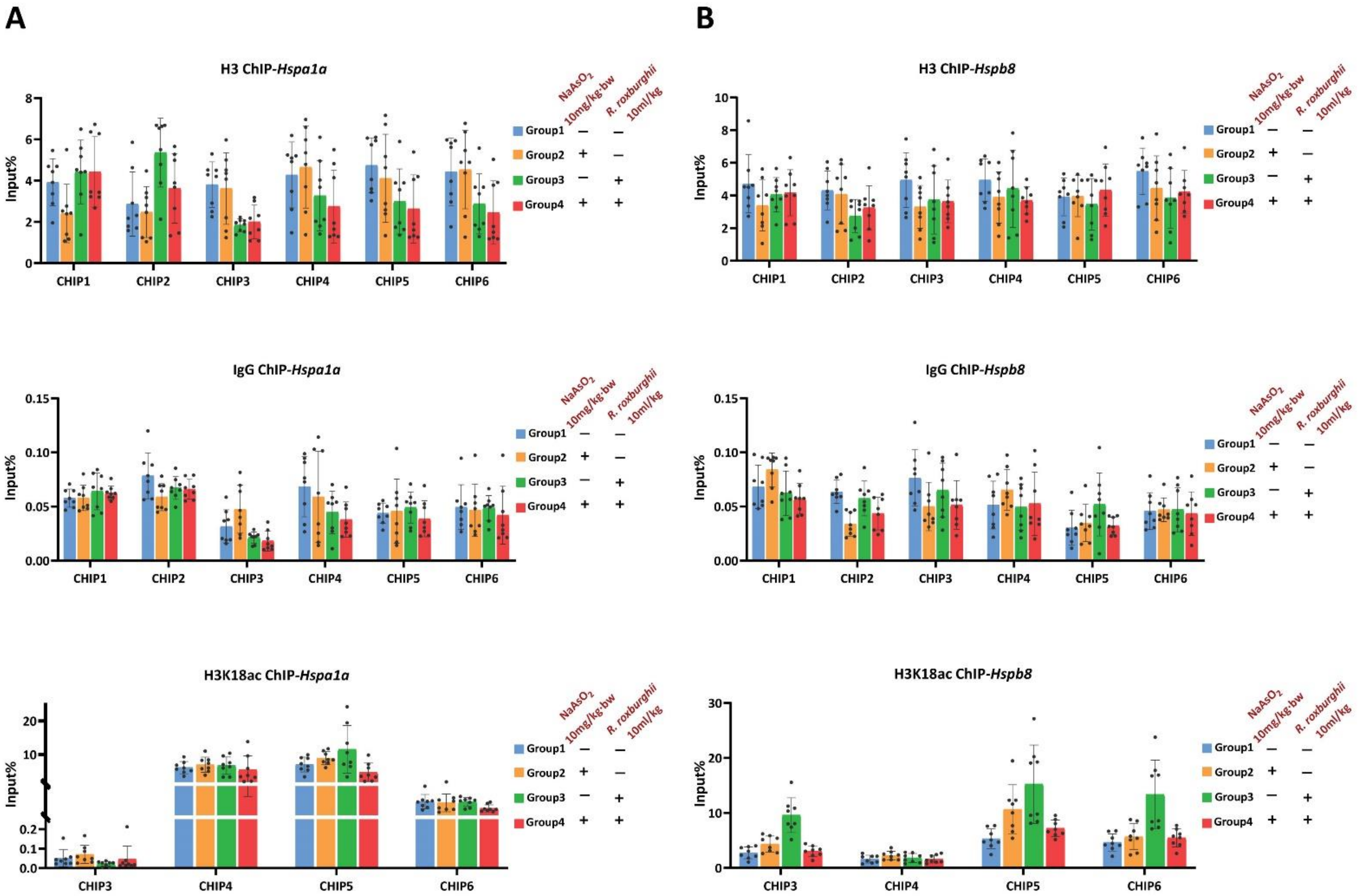

Figure S3 The enrichment of H3, IgG and H3K18ac in promoters of *Hspa1a* (A) and *Hspb1* (B) genes in liver of rats in controls, arsenic group (10.0 mg/kg·bw $NaAsO_2$), single *R. roxburghii* juice group (10 ml/kg *R. roxburghii* juice) and *R. roxburghii* juice antagonist group (10.0 mg/kg·bw $NaAsO_2$ + 10 ml/kg *R. roxburghii* juice).

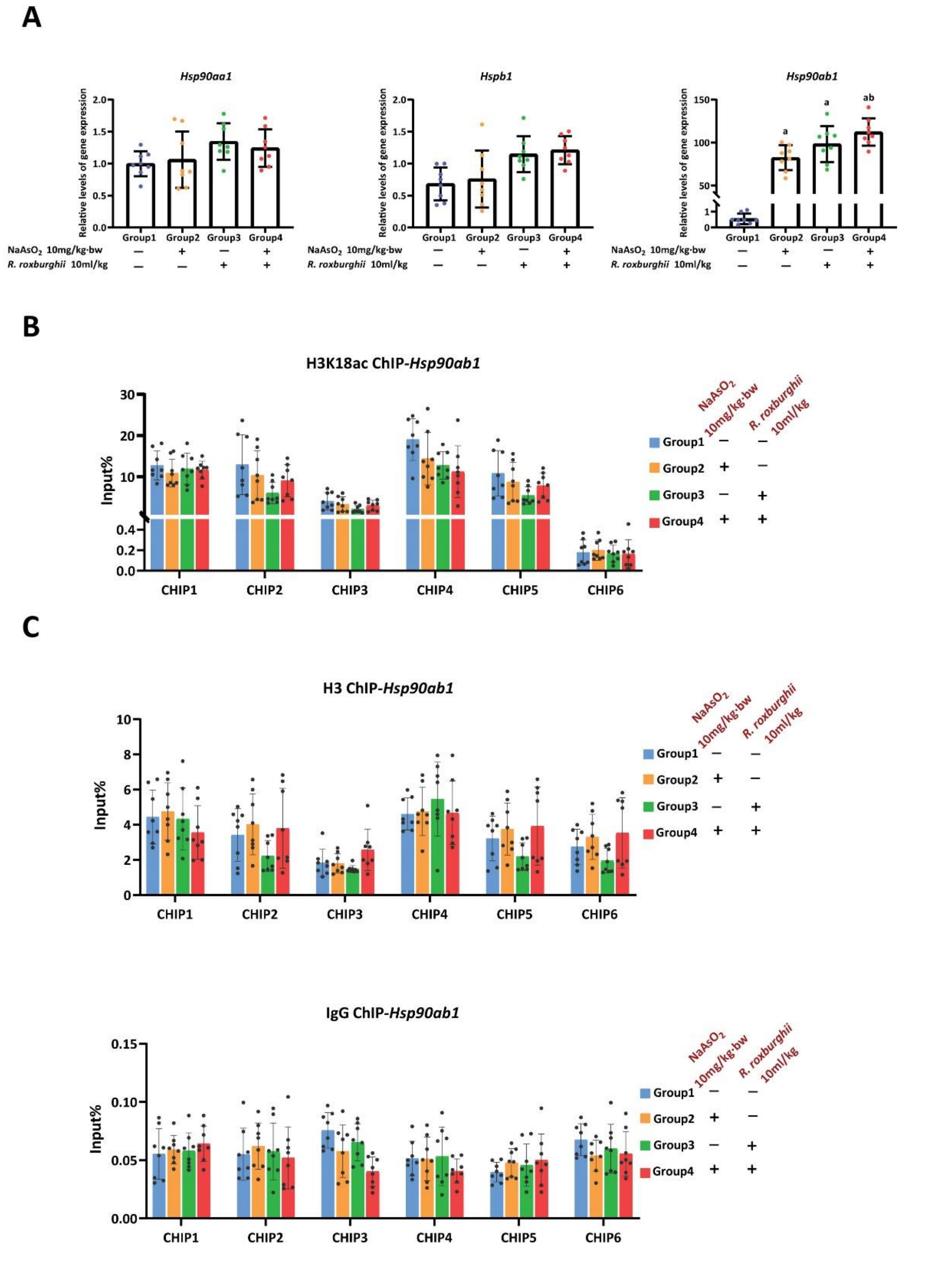

Figure S4 Expression of *Hsp90aa1, Hspb1 and Hsp90ab1* (A) and the enrichment of H3K18ac (B), H3 and IgG (C) in the gene promoters of rats' liver in controls, arsenic group (10.0 $NaAsO_2$ mg/kg·bw), single *R. roxburghii* juice group (10 ml/kg *R. roxburghii* juice) and *R. roxburghii* juice antagonist group (10.0 mg/kg·bw $NaAsO_2$ + 10 ml/kg *R. roxburghii* juice). In (A), a, b represents $P<0.05$ compared with controls and arsenic group, respectively.
